# A model of lightness perception guided by probabilistic assumptions about lighting and reflectance

**DOI:** 10.1167/jov.20.7.28

**Published:** 2020-07-29

**Authors:** Richard F. Murray

**Affiliations:** Department of Psychology and Centre for Vision Research, York University, Toronto, Ontario, Canada

**Keywords:** lightness, illumination, computational modeling, psychophysics

## Abstract

Lightness perception is the ability to perceive black, white, and gray surface colors in a wide range of lighting conditions and contexts. This ability is fundamental for any biological or artificial visual system, but it poses a difficult computational problem, and how the human visual system computes lightness is not well understood. Here I show that several key phenomena in lightness perception can be explained by a probabilistic graphical model that makes a few simple assumptions about local patterns of lighting and reflectance, and infers globally optimal interpretations of stimulus images. Like human observers, the model exhibits partial lightness constancy, codetermination, contrast, glow, and articulation effects. It also arrives at human-like interpretations of strong lightness illusions that have challenged previous models. The model's assumptions are reasonable and generic, including, for example, that lighting intensity spans a much wider range than surface reflectance and that shadow boundaries tend to be straighter than reflectance edges. Thus, a probabilistic model based on simple assumptions about lighting and reflectance gives a good computational account of lightness perception over a wide range of conditions. This work also shows how graphical models can be extended to develop more powerful models of constancy that incorporate features such color and depth.

## Introduction

We perceive the colors of objects effortlessly and for the most part reliably, even though light intensity at the retina provides highly ambiguous information about surface properties (Land and McCann, 1971; Belhumeur et al., 1999). Understanding how the visual system overcomes this ambiguity to compute lightness (i.e., perceived surface reflectance^1^) is a difficult problem with a long history (Hering, 1905/1964; von Helmholtz, 1910/1924). Research over several decades has shown, however, that features such as perceived lighting boundaries, depth discontinuities, and cues to transparency play a central role in lightness perception; for reviews, see Adelson (2000), Gilchrist (2006), and Kingdom (2011).

To take just one example, Gilchrist et al. (1999) have explored the role of lighting boundaries and formulated an “anchoring theory” that consists of several principles of lightness perception. The “highest luminance rule” states that the highest-luminance element in a perceived lighting region is assigned a local perceived reflectance value of 0.90. The principle of “codetermination” states that an element's perceived lightness depends not only on its lightness assignment in its local lighting region, but also on its lightness assignment in a larger, global region. “Scale normalization” states that the visual system tends to expand the range of perceived lightness values in a scene towards the full range from black to white. These principles and others provide a broad understanding of how we perceive lightness under a wide range of conditions.

However, theories of lightness have generally not been formulated as computational models. Anchoring theory, for example, although able to make quantitative predictions for some stimuli (Economou et al., 2007), nevertheless relies on the modeler to identify perceived lighting boundaries and specify their strength. The fact that theories of lightness are usually not computational means that they cannot make clear predictions for many stimuli, and it also limits their usefulness in applications. This stands in sharp contrast to theories of brightness (i.e., perceived luminance^1^), which are almost always image-based computational models, and can make falsifiable predictions for any achromatic two-dimensional stimulus (e.g., Blakeslee and McCourt, 1999; Dakin and Bex, 2003).

Still, there have been a few computational theories of lightness. Land and McCann's (1971) retinex theory estimates reflectance by discounting illumination at sharp boundaries, although more recent versions of retinex have aimed at modeling image “appearance” instead of lightness (McCann and Rizzi, 2012, chapter 12). Adelson and Pentland (1996) described an optimization approach to recovering lighting, shape, and reflectance in polyhedral scenes. Allred and Brainard (2013) developed a Bayesian algorithm for predicting lighting and reflectance percepts in a 5 × 5 grid stimuli, and tested this model against psychophysical data. And although not a theory of human lightness perception, Barron and Malik (2015) developed a computer-vision algorithm for estimating lighting, shape, and reflectance that is a useful point of comparison for theories of human vision. All of these theories have been, to varying extents, motivated by observations about statistical properties of lighting and reflectance in natural scenes. Exploiting natural scene statistics provides a principled approach to overcoming the intrinsic ambiguity of retinal images, and potentially even an optimal approach (Geisler, 2008). This is the approach that I follow in the present work as well.

## Some probabilistic modelling tools

The lightness model that I describe below is a conditional random field (CRF), which is a type of Markov random field (MRF). I cannot give an adequate introduction to CRFs, MRFs, and probabilistic graphical models here, but in the following paragraphs I outline some key concepts so that readers unfamiliar with these tools can understand the gist of the lightness model. More complete introductions can be found in Koller and Friedman (2009), Bishop (2006), and Prince (2012).

In many statistical modeling problems, there is a large number of individual elements, and each element has statistical dependencies on many other elements. An MRF model makes the simplifying assumption that each element has a small number of “neighbors,” and that all statistical relationships are byproducts of statistical relationships between neighboring elements. More precisely, each element of an MRF is conditionally independent of its non-neighbors, given the state of its neighbors. In a model of natural images, for example, we could adopt a model where each image pixel is a neighbor of the eight immediately adjacent pixels. Distant image pixels may be correlated, but in this example the MRF model posits that these long-range correlations are consequences of local statistical relationships between neighboring pixels.

The probability density function of an MRF can be written in the following form,^2^ called a “Gibbs distribution”:

(1) $$P(X)=\frac{1}{Z}\;\text{exp}\left(-\sum_{i}\varepsilon_{\;i}\left(C_{i}\right)\right)$$

A “clique” is a set of elements in which all pairs are neighbors, and here the sum is over all cliques *C*_*i*_ of the MRF. In the natural image example where each pixel has eight neighbors, a little thought shows that the largest cliques are 2 × 2 squares of pixels. The functions ϵ_*i*_ are “potential functions” that put a cost on the state of each clique, such that states with high costs tend to occur less frequently^3^. *Z* is a constant that gives the density function a total volume of 1. The probability density of a state *X* of the ensemble is determined by the the sum of the potentials of all cliques under that state. Thus, we can specify the probability density of an MRF by specifying a set of potential functions ϵ_*i*_ on cliques, and because cliques tend to be small, this makes modeling the ensemble of elements much more tractable.

In some modeling problems there are “observed” elements, whose states we know, and “hidden” elements, whose states we wish to infer. In shape from shading, for example, the observed elements may be the pixels of a stimulus image, and the hidden elements may be variables that give the slant and tilt of a surface at each pixel location. A CRF is a variant of the MRF that is useful for such problems (Lafferty & McCallum, 2001). In a CRF, the potential functions ϵ_*i*_ for hidden elements depend on the state of the observed elements. In the shape-from-shading example, we observe a stimulus image, and a CRF model would then provide a corresponding set of potential functions ϵ_*i*_ that specify (as in Equation 1) the probability density of slant and tilt across the image. We then interpret Equation 1 as the conditional probability density of the hidden elements, given the observed elements.

Even when a CRF provides a probability density over hidden variables, making a point estimate of those variables, such as a maximum a posteriori (MAP) estimate, can be a difficult problem. The lightness model described here addresses this problem using a belief propagation algorithm. Belief propagation is a method where elements are grouped into clusters, and each cluster passes “messages” to clusters that overlap with it; in “max-sum” belief propagation, the method used here, the message contains information about the most probable state of the elements that the sender and receiver have in common. As messages are passed, information about the most probable states of elements is propagated through the ensemble. If the message passing network has no loops, then belief propagation is guaranteed to find the most probable joint state of the elements. If the network has loops (as in the lightness model described elsewhere in this article) then the algorithm is not guaranteed to find the most probable state, or even to converge. Nevertheless, in practice belief propagation has been found to work well even in many problems where the message passing network has loops.

## A probabilistic model of lightness perception

**Figure 1.**
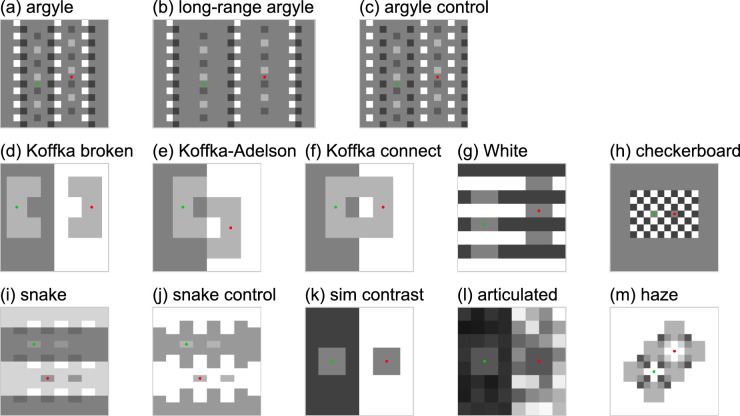
Grid figures. The red and green dot locations have the same reflectance within each figure. (a) Argyle illusion (Adelson, 1993). (b) Long-range argyle illusion (Flynn & Shapiro, 2014). (c) Argyle control figure (Adelson, 1993). (d) Koffka ring, broken (Koffka, 1935). (e) Koffka-Adelson figure (Adelson, 2000). (f) Koffka ring, connected (Koffka, 1935). (g) White's illusion (White, 1979). (h) Checkerboard assimilation (DeValois and DeValois, 1988). (i) Snake illusion (Adelson, 2000). (j) Snake control figure (Adelson, 2000). (k) Classic simultaneous contrast figure (Hess & Pretori, 1894/1970). (l) Articulated simultaneous contrast figure (Katz, 1935). (m) Haze illusion (Adelson, 2000).

My goal in this article is to describe a model that produces qualitatively human-like lightness judgments with a wide range of stimuli. To focus on some of the problem's essential features, I model stimuli on a simple 16 × 16 grid. Within this constraint, one can create many phenomena that challenge current theories, and a thorough account of this domain would explain a great deal about lightness perception. For example, Figure 1 illustrates several lightness phenomena on a 16 × 16 grid, including effects of perceived lighting boundaries (Figure 1a), simultaneous contrast (Figure 1k), articulation (Figure 1 l), assimilation (Figure 1g), and translucency (Figure 1m). No current computational model can account for all these effects.

**Figure 2.**
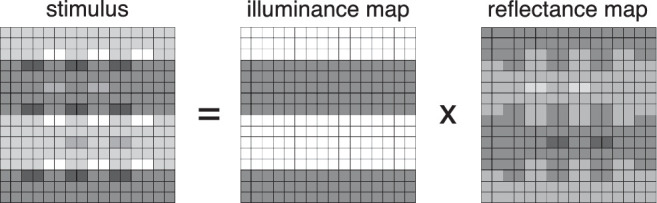
Layer structure of the MIR model. The luminance stimulus is represented as a pointwise product of illuminance and reflectance maps. Local statistical assumptions about 2 × 2 patches of illuminance and reflectance guide the model in estimating the illuminance and reflectance maps. The illuminance and reflectance maps shown here are illustrative; see Figures 5 and 6 for model results.

The lightness model I propose is guided by a local statistical model of lighting and reflectance. The model is a CRF that uses statistical assumptions about small image patches to infer globally optimal estimates of lighting and reflectance. I call the model MIR, for “Markov illuminance and reflectance.” The model has three layers: a reflectance map, an illuminance map that represents incident lighting magnitude,^1^ and a luminance map that represents the observed stimulus (Figure 2). Given a luminance map, the model constructs potential functions on 2 × 2 patches of the illuminance and reflectance maps. The potential functions impose the following soft constraints. (a) Reflectance mostly spans the range 3% to 90%, with a rapid decline in probability outside these limits. (b) Low illuminances are more probable than high illuminances. (c) Illuminance edges are less common than reflectance edges. (d) Reflectance and illuminance edges usually occur at image luminance edges (Freeman, 1996). (e) X-junctions are evidence for illuminance edges (Metelli, 1970; Beck et al., 1984). (f) Costs are evaluated on uniform image regions instead of pixelwise^4^ (Katz, 1935; Gilchrist, 2006). (g) Illuminance edges tend to be straighter than reflectance edges (Logvinenko et al., 2005). Given a stimulus image, MIR uses belief propagation to find global illuminance and reflectance assignments that generate the image and match these local assumptions as closely as possible. I describe these assumptions quantitatively in the Appendix, and I provide a MATLAB implementation of the model at doi:10.17605/OSF.IO/4FWJV.

These assumptions are tentative, and I have chosen them because either (a) they reflect simple properties of lighting and reflectance or (b) they are suggested by previous literature, and together they explain a range of phenomena in lightness perception. In the general discussion section, I discuss possible alternative assumptions, and alternative ways of arriving at the assumptions that drive the model.

## Experiment 1

**Figure 3.**
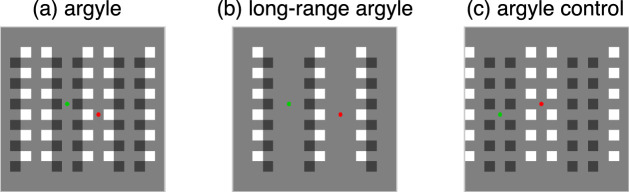
Argyle stimuli in Experiment 1. Each image is physically the same at the green and red dot locations. (a) Argyle illusion. (b) Long-range argyle illusion. (c) Argyle control figure.

To obtain lightness data that can be used to test MIR and related models, I ran a behavioral experiment using several grid stimuli. I tested whether people perceive the expected illusions in the stimuli shown in Figures 1 and 3, which are adaptations of well-known lightness illusions for a 16 × 16 grid. The first part of the experiment tested for lightness illusions: observers viewed reflectance-calibrated printouts of grid stimuli under uniform lighting and judged which of two isoluminant target regions was a lighter shade of printed gray. The second part tested the relative strength of lightness illusions: observers viewed pairs of grid stimuli, and judged which had a greater lightness difference between the two isoluminant target regions.

### Methods

#### Observers

There were 20 participants, recruited from York University and paid $25. All procedures were approved by the Office of Research Ethics at York University and adhere to the Declaration of Helsinki.

#### Stimuli

The stimuli were 8-cm square printed paper images. A Konica Minolta LS-110 photometer (Konika Minolta, Tokyo, Japan) and a Labsphere Spectralon 99% diffuse reflectance standard (model SRS-99-020; Labsphere, North Sutton, NH) were used to characterize the mapping from gray level to reflectance for an HP Color LaserJet Pro printer (model M452dw; HP Inc., Palo Alto, CA). This mapping was used to print the required reflectance patterns on white letter paper (reflectance 82%), and then the stimulus figures were cut out from these prints. The stimulus set included the following images from Figure 1: broken Koffka ring, Koffka-Adelson figure, connected Koffa ring, White's illusion, checkerboard assimilation figure, snake illusion, snake control, simultaneous contrast, and articulated simultaneous contrast. It also included the argyle, long-range argyle, and argyle control images from Figure 3. It did not include the haze illusion, which does not produce a simple difference in perceived reflectance between the two target locations. The stimulus set included a left-right, mirror-reversed version of each image, so that any left-right response biases would not cause observers to choose one target region more often than the other. Two small green dots (diameter 0.4 mm) in each figure indicated the two target regions where observers were to judge lightness. (In Figure 1 and elsewhere in this article I use red and green dots so that I can refer to the target regions separately, but in the experiment both dots were green.) The paper figures were shown in a room illuminated by overhead fluorescent lights, and the white regions of the stimuli (reflectance 82%) had a luminance of 89 cd/m^2^. The 16 × 16 reflectance patterns are provided with the model code at doi:10.17605/OSF.IO/4FWJV.

#### Procedure

Each observer participated in one 10-minute session. In the first part of the session, the observer sat at a viewing distance of 57 cm from a small wooden stand on a table covered by a matte black cloth. Head position was stabilized with a chin rest. On each trial, the experimenter placed a paper stimulus figure on the wooden stand. The 24 stimuli were shown in a different random order for each observer, with the constraint that a stimulus and its mirror reversal were separated by at least two intervening trials. Each stimulus was 8 cm square and subtended 8° of visual angle. From the observer's viewing position, the wooden stand was occluded by the stimulus (the back of each paper figure had a small attachment that the experimenter latched onto the stand), so there was a depth discontinuity at the edges of the paper figure; previous work shows that depth discontinuities tend to make a region function as an independent lighting framework (Gilchrist, 2006). The observer responded “left” or “right” to indicate which of two equiluminant target regions in the stimulus, indicated by small green dots, seemed to be printed with a lighter shade of grey ink. The observer was also given the choice of responding “same” if they did not perceive a difference between the printed grays of the two target regions. Trials where observers responded “same” were counted as a 0.5 trial contribution to both the “left” and “right” response counts. The observer typically took between 0.5 and 2 seconds to make a response. The experimenter recorded the response, removed the figure, and began the next trial.

In the second part of the session, which immediately followed the first, the observer sat at a viewing distance of 57 cm from two small wooden stands. On each trial, the experimenter placed a paper stimulus figure on each stand. The pairs of stimuli were: argyle illusion and argyle control; long-range argyle illusion and argyle control; argyle illusion and simultaneous contrast; snake illusion and snake control; broken Koffka ring and connected Koffka ring; Koffka-Adelson figure and connected Koffka ring; and simultaneous contrast and articulated simultaneous contrast. Each pair was shown on two trials, once with the first figure on the left and the second on the right, and once with the opposite placement. The 14 resulting pairs were shown in a different random order for each observer, with the constraint that each pair and its left-right opposite-placement pair were separated by at least two intervening trials. The observer responded “left” or “right” to indicate which figure had target regions that differed more in their printed shades of gray. The observer could also respond ‘same’ if the two figures seemed to have the same difference between their target regions. The experimenter recorded the response, removed the stimuli, and began the next trial. Observers’ trial-by-trial responses are available at doi:10.17605/OSF.IO/4FWJV.

### Results and discussion

**Figure 4.**
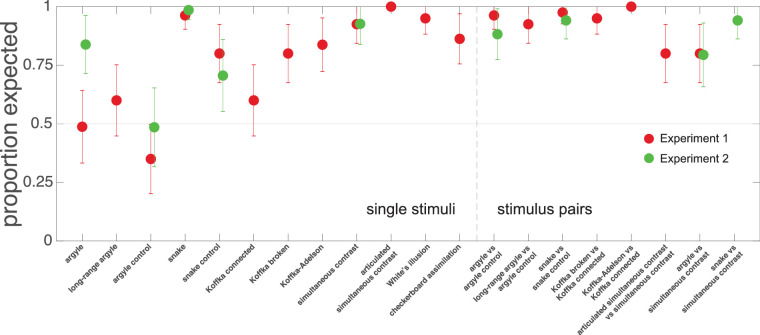
Results of Experiments 1 and 2. Data points to the left of the vertical dashed line show results from trials where observers judged the relative lightness of two target patches in a single figure. Data points to the right show results from trials where observers judged which of two figures had the greater lightness difference between two target patches. Each data point shows the proportion of trials on which observers chose the expected target patch or the expected figure (see text for details). Error bars show 95% confidence intervals. The two experiments used different subsets of the stimulus figures, so not all conditions have both red and green data points. Furthermore, Experiments 1 and 2 used different stimuli in the argyle and argyle control conditions.

The left side of Figure 4 shows results from the first part of the experiment, where one stimulus was shown on each trial. Based on the previous literature, observers were expected to choose the leftmost target region in each stimulus in Figures 1 and 3. (Recall that the experiment also included the left-right reversal of each stimulus; in those stimuli observers were expected to choose the rightmost target region.) Observers saw the expected lightness illusion in most of the stimuli (Figure 4). They chose the expected target region at significantly above-chance rates with the snake, snake control, broken Koffka, Koffka-Adelson, simultaneous contrast, articulated simultaneous contrast, White, and checkerboard assimilation stimuli. Observers did not choose the expected target region at significantly above-chance rates with the connected Koffka or argyle control figures, which are known to create weak or nonexistent lightness illusions, and are typically used as control figures.

However, observers also did not choose the expected target region at significantly above-chance rates with the argyle or long-range argyle stimuli. In their original form, these lightness illusions are strong and reliable, so these findings show that the grid figures did not recreate the illusions adequately. Interestingly, the problem was not that the target regions in these figures seemed to have similar shades of gray. Observers usually judged the target regions to have a greater lightness difference in the argyle figure than in the simultaneous contrast figure (Figure 4, right-hand side), and they reliably perceived the expected illusion in the simultaneous contrast figure (Figure 4, left-hand side). Furthermore, after the experiment was finished I asked some observers informally how they perceived the stimuli and found that they typically experienced strong lightness illusions with the argyle and long-range argyle figures, but some saw the illusion in the expected direction and others saw it in the opposite direction. As in reports of #thedress (Brainard & Hurlbert, 2015; Murray & Adams, 2019), observers experienced a strong illusion and found it difficult to believe that others saw the figure differently. These individual differences may be related to the perceived illumination pattern in the grid argyle figures, where the target patch that some observers see as a darker shade of gray (as expected) also appears to be in a brighter lighting region. In any case, the models tested here do not address individual differences, so these versions of the argyle and long-range argyle illusions are not useful for model testing. Experiment 2 tested a revised version of the argyle figure.

The right side of Figure 4 shows results from the second part of the experiment, where two stimuli were shown on each trial. For all stimulus pairs, observers chose the stimulus that we would expect from the previous literature to have the stronger lightness illusion, at rates significantly above chance. The argyle illusion and long-range argyle illusion were chosen more often than the argyle control figure. The snake illusion was chosen more often than the snake control figure. The broken Koffka ring and Koffka-Adelson figures were chosen more often than the connected Koffka ring. The argyle and snake figures, typically strong lightness illusions, were chosen more often than the simultaneous contrast figure. And the articulated simultaneous contrast figure was chosen more often than the standard simultaneous contrast figure.

## Experiment 2

Experiment 2 repeated Experiment 1 with a subset of the stimuli, as well as a revised grid argyle figure with stronger articulation and grouping cues in perceived lighting regions. As shown in Figure 1a, the revised argyle figure has boundaries that extend all the way to the edge of the stimulus (presumably segmenting the perceived lighting regions more strongly), and has light and dark squares in the two central perceived lighting regions (which increases articulation).

### Methods

#### Observers

There were 17 participants, recruited from York University and paid $15. Eleven had participated in Experiment 1.

#### Stimuli

The stimuli were the following images from Figure 1: argyle illusion, argyle control, snake illusion, snake control, and simultaneous contrast.

#### Procedure

The procedure was the same as in Experiment 1. The session took approximately 3 minutes. In the first part of the session, the observer viewed a single stimulus on each trial, and judged which of two target regions was a lighter shade of printed gray. In the second part, the observer viewed the following pairs: argyle illusion and argyle control; argyle illusion and simultaneous contrast; snake illusion and snake control; and snake illusion and simultaneous contrast. The observer judged which figure had a greater lighter difference between the two target regions. As in Experiment 1, the stimulus set included the left-right mirror reversal of each figure, and the left-right opposite placement of each pair of figures.

### Results and discussion

The green data points in Figure 4 show the proportion of trials on which observers chose the expected target region or stimulus. The left side of Figure 4 shows that observers saw the expected lightness illusions. In particular, observers saw the expected illusion in the revised argyle figure, but little or no illusion in the revised argyle control figure. The right side of Figure 4 shows that observers chose the figure that we would expect from previous literature to have the stronger lightness illusion, at rates significantly above chance.

## Model results: lightness illusions

**Figure 5.**
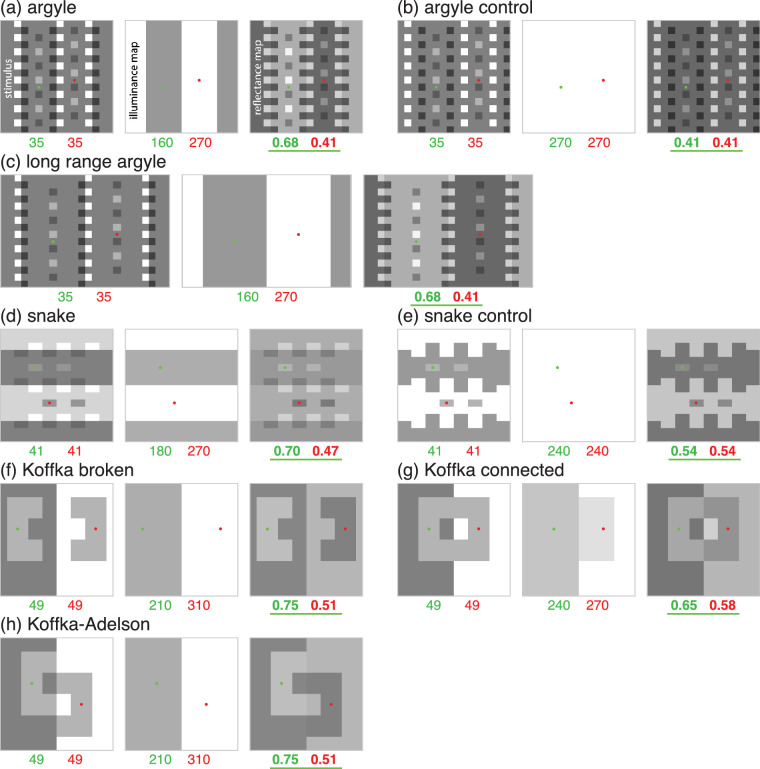
MIR's responses to lightness illusions. In each lettered panel, the left image shows the stimulus, the middle image shows MIR's illuminance map output, and the right image shows MIR's reflectance map output. Red and green dots in the stimulus images show isoluminant target locations where human observers judged reflectance. Red and green dots are also shown at corresponding locations in the model output images for reference. Red and green numbers show the model's outputs at those locations (stimulus luminance units are cd/m^2^, illuminance map units are lux). Human observers judged the green target locations to be as light as or lighter than the red target locations at rates significantly above chance (see Figure 4). The model's reflectance outputs (in bold and underlined) correctly predict a strong lightness illusion in stimuli a, c, d, f, and h, and not in stimuli b, e, and g. One caveat is that the model predicts no illusion in the snake control stimulus (e), whereas human observers see a weak contrast effect. As in the perceptual experiments, the stimulus set did not include the haze illusion because it does not produce a simple lightness difference between the two target locations. See Figure 6 for further results.

**Figure 6.**
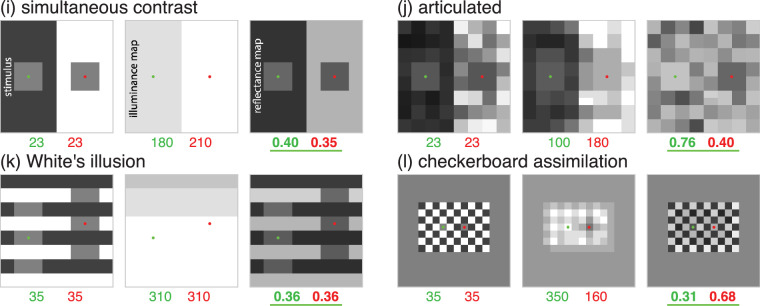
MIR's responses to additional lightness illusions. MIR correctly predicts a contrast effect in stimulus i, and a stronger contrast effect in stimulus j. MIR does not correctly predict the assimilation effects in stimuli k and l. See caption of Figure 5 for details.

I tested the MIR lightness model on the same stimuli shown to human observers in Experiments 1 and 2 (Figure 1), except that I did not use the version of the argyle figures that resulted in large individual differences in Experiment 1. I took MIR's reflectance output at the two isoluminant test locations to be its prediction of human lightness percepts for the grid stimuli. The test was qualitative: If the model produced a higher lightness response for the target region that human observers chose significantly more often as appearing lighter, then the model was judged to have predicted human observers’ responses correctly for that stimulus. The results are shown in Figures 5 and 6, and summarized in Table 1.

**Figure 7.**
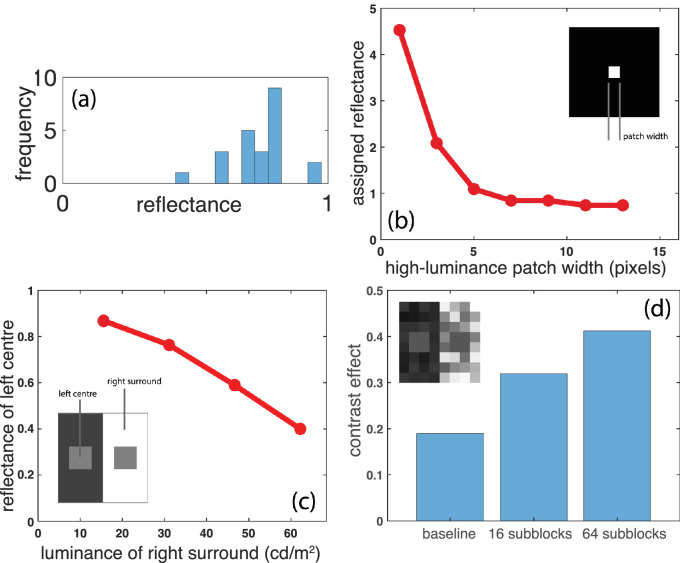
Lightness phenomena. (a) Highest luminance rule: MIR assigns a white reflectance (around 0.8) to the highest luminance in each region of the stimuli in Figure 1 that it interprets as a strongly segmented lighting region (see the illuminance maps in Figures 5 and 6 for assigned lighting regions). (b) Glow: A small luminance outlier is assigned a reflectance greater than one, but as the width of the region increases, the assigned reflectance decreases to around 0.8. (c) Codetermination: In the classic simultaneous contrast figure, the reflectance that MIR assigns to the left-hand center patch decreases as the luminance of the right-hand surround increases. (d) Articulation: As the stimulus is subdivided into more regions, the simultaneous contrast effect (i.e., the difference between the reflectances assigned to the two center patches) increases. The ‘baseline’ stimulus is the classic simultaneous contrast image shown in Figure 1k, and the inset shows the 64 subblock stimulus.

**Table 1.** Model test results. A check mark indicates that the model qualitatively predicts the lightness illusion seen by human observers (for single stimuli) or the relative strength of two illusions (for stimulus pairs). An x indicates that it does not.

	MIR	ODOG	High-pass	Retinex
Single stimuli				
Argyle	√	√	√	√
Broken argyle	√	√	√	√
Snake	√	√	√	√
Snake control	×	√	√	√
Koffka broken	√	√	√	√
Koffka-Adelson	√	√	√	√
Koffka connected	√	√	√	√
Simultaneous contrast	√	√	√	√
Articulated Simultaneous contrast	√	√	√	√
White's illusion	×	√	×	×
Checkerboard assimilation	×	√	×	×
Stimulus pairs				
Argyle vs. broken	√	×	×	×
Snake vs. control	√	√	√	√
Koffka broken vs. Koffka connected	√	√	×	×
Koffka-Adelson vs. Koffka connected	√	×	×	×
Articulated vs. simultaneous contrast	√	×	×	√
Argyle vs. simultaneous contrast	√	×	×	√
Snake vs. simultaneous contrast	√	×	√	×

Like human observers, MIR interprets the argyle figure as having bright and dark vertical strips of lighting (Figure 5a). The model takes these lighting estimates into account when computing reflectance, and so image locations that have the same luminance are assigned very different reflectances. Also like human observers, the model interprets the argyle control figure as having uniform lighting (Figure 5b), so in this case it assigns the same reflectance to the two isoluminant target patches.

The model also makes qualitatively human-like interpretations of most of the other stimulus figures. Although the model makes only local statistical assumptions on 2 × 2 image patches, belief propagation distributes local information broadly, so the model interprets the long-range argyle illusion much like the standard argyle (Figure 5c). The model predicts a strong lightness difference in the snake illusion and not in its control condition (Figure 5d, e). The Koffka ring figures depend on the presence or absence of a perceived lighting boundary, and the model makes human-like assignments here as well (Figure 5f, g, h). The model accounts for classic and articulated simultaneous contrast (Figure 6i, j), even though it has no explicit mechanism for lateral inhibition (Economou et al., 2007; Ratliff, 1965). (MIR predicts a lightness illusion in the classic simultaneous contrast figure, but not in the snake control figure, because in the snake control the long boundaries that span the image have corners, and the model places a high cost on lighting boundaries that have corners. Thus, in the simultaneous contrast figure the long boundary is interpreted as a partial illuminance edge, whereas in the snake control it is interpreted purely as a reflectance edge.)

Furthermore, the model accounts for the relative strength of lightness illusions in all stimulus pairs compared by human observers in Experiments 1 and 2 (see summary in Table 1). The model correctly predicts stronger lightness illusions in the argyle, snake, broken Koffka, and Koffka-Adelson figures than in their control conditions. It also correctly predicts stronger illusions in the argyle, snake, and articulated figures than in the classic simultaneous contrast figure.

One limitation of the model is that it does not account for assimilation effects, as in White's illusion and the checkerboard assimilation stimulus (Figure 6k, l). Assimilation is a challenging phenomenon for lightness models like MIR that rely on discounting illumination, because it shows that surrounding a target patch with high-luminance elements, which we would generally expect to *increase* the illumination estimate, can cause the target patch to appear lighter, not darker. One possibility for a future revision of MIR is to incorporate a prior for smooth reflectance patterns, which would make each reflectance value tend to be similar to surrounding reflectance values.

## Model results: lightness phenomena

In addition to accounting for these illusions, MIR also produces and explains several key phenomena in lightness perception. The highest luminance rule, for example, states that the highest luminance in a lighting region tends to be seen as white (Wallach, 1948; Land & McCann, 1971; Gilchrist, 2006). Figure 7a shows that the model assigns a high reflectance to the highest luminance in each inferred lighting region in Figures 5 and 6. This behavior follows naturally from the model's goal of assigning low illuminances while mostly limiting reflectance to the range of 3% to 90%, which implies that the highest luminance in a strongly segmented lighting region will tend to have a reflectance of around 90% (Murray, 2013). To generate the lightness values histogrammed in Figure 7a, I found the highest reflectance estimate in each uniform illuminance region, for example, in Figure 5a there are four vertical strips in the illuminance map, so I found the highest reflectance in each of the four corresponding regions of the reflectance map.

**Figure 8.**
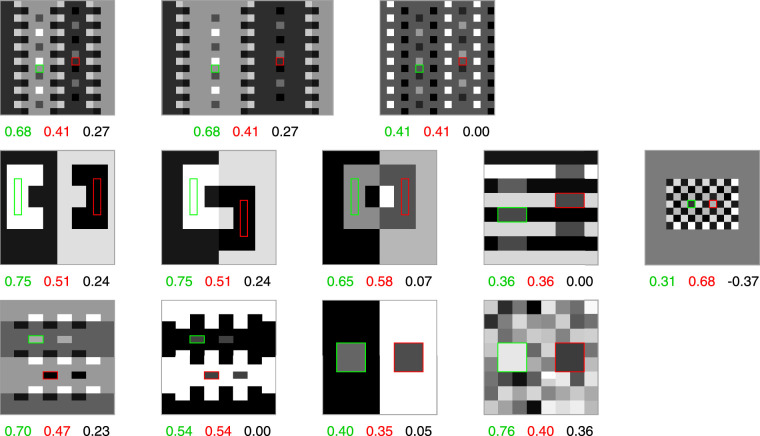
MIR results. The green and red values below each panel show the model's mean reflectance output inside the green and red outlined target regions, respectively. These are the same target regions judged by human observers in the perceptual experiments. The value in black shows the difference between the two values (green minus red). A positive value in black indicates a correct qualitative prediction of the illusion seen by human observers.

Sometimes, however, the highest luminance in a scene does not appear to be a reflective white, but instead appears to glow. MIR accounts for this phenomenon as well (Figure 7b). The model assumes that reflectance mostly spans the range of 3% to 90%, but that with some small probability it can extend above 90%, and even above 100%, which is one way of representing glow (see the Appendix for details; Bonato & Gilchrist, 1994). The model's potential functions imply that assigning a moderate reflectance to a small luminance outlier is not worth the cost of either positing a strong illuminance edge or assigning a high illuminance to the whole figure, so instead the model posits a weak illuminance edge and assigns the outlier a reflectance greater than 100%. A large luminance outlier is worth the cost, however, and so the model is less likely to see large regions as glowing (Figure 7b). This is also true of human observers (Bonato and Gilchrist, 1999).

Codetermination refers to the fact that the perceived reflectance of an image patch often depends not only on the image luminances in its own lighting region, but also on adjacent lighting regions (Kardos, 1934; Gilchrist, 2006). MIR shows codetermination as well (Figure 7c). The model uses a smooth cost function that often interprets luminance edges as a combination of illuminance and reflectance edges (i.e., partial lighting boundaries), rather than making all-or-none assignments. At a partial lighting boundary, stimulus features that affect perceived illuminance on one side of the boundary will generally affect perceived illuminance on the other side as well, and so luminance values in one lighting region affect the model's reflectance estimates in adjacent regions.

Lightness contrast effects are often stronger when an image is “articulated,” that is, composed of several distinct luminance regions (Katz, 1935). MIR also shows articulation effects (Figure 7d). The model produces simultaneous contrast, and this contrast effect is stronger in articulated figures. (The articulated stimuli are generated by randomly perturbing the luminance of each subblock. Each bar shows the median simultaneous contrast effect over 100 stimulus samples.) This articulation effect occurs because the model's potentials are evaluated on uniform luminance regions,^4^ not pixelwise, so when the image is divided into a larger number of uniform regions there is a greater cost reduction for positing a strong lighting boundary along the vertical edge that divides the figure in two.

Significantly, these model behaviors do not depend on fine-tuned assumptions, and are generated by simple cost functions such as a logarithmic cost on illuminance and a sum-of-squares cost on illuminance edges (see the Appendix for details). MIR shows how several broad features of human lightness perception, which might seem to be idiosyncratic and unrelated, follow naturally from a few generic, probabilistic assumptions about lighting and reflectance.

## Comparison to other models

Theories of *brightness* (i.e., perceived luminance^1^) are typically computational models that use operations such as spatial filtering and contrast normalization derived from the physiology of early visual cortical areas (DeValois & DeValois, 1988), or are based on simple stimulus properties such as luminance ratios at edges (Heinemann, 1955; Rudd & Zemach, 2004). The relationship between lightness and brightness is not well understood, partly because of this difference between the goals and methods of the researchers who study them. Nevertheless, under some viewing conditions we expect lightness and brightness judgments to be similar, for example, with simple, reflective, uniformly illuminated two-dimensional geometric figures such as the ones used in Experiments 1 and 2, for which luminance is proportional to reflectance. Thus, with some caveats it can be useful to evaluate models of lightness and brightness on the same stimuli, as has often been done in the past (Adelson, 1993; Shapiro & Lu, 2011; Blakeslee & McCourt, 2012).

I compared the results with MIR on grid images to three current computational models: Oriented Difference of Gaussians (ODOG) (Blakeslee & McCourt, 1999), a high-pass model (Shapiro & Lu, 2011), and a retinex model (Land & McCann, 1971; McCann, 1999). I provide MATLAB implementations of these models and code to run them on the grid stimuli used here at doi:10.17605/OSF.IO/4FWJV. For easier comparison, Figure 8 shows MIR's reflectance outputs from Figures 5 and 6, reorganized into the format used below for the other three models. Usually, I evaluate models on both an illusion and its control condition. I report the model as having “accounted for” an illusion if it predicts the expected illusion, but by itself this is too liberal a criterion: If the model also predicts an illusion in the control condition, then it cannot really be said to have explained the illusion. Thus, in what follows I will draw attention to each model's performance both with illusions and also with their control conditions.

**Figure 9.**
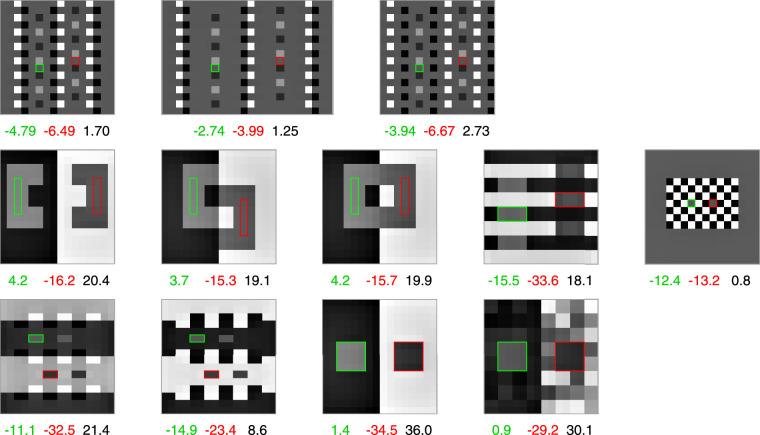
ODOG results. The images shown here are downsampled to 16 × 16 pixels for easier comparison with the results from MIR (Figure 8). Each pixel shows the average value in the corresponding region of the full-resolution (1024 × 1024 pixel) model output. See caption of Figure 8 for details.

ODOG is a spatial filtering model of brightness (Blakeslee & McCourt, 1999). It is based on the responses of oriented filters at several orientations and scales, and also incorporates a response normalization stage. To test this model, I used a MATLAB translation of the Mathematica code in Blakeslee et al. (2016). The model inputs were the grid stimuli shown in Figure 1 (except for the haze illusion), upsampled to 512 × 512 pixels (except for the long-range argyle, which was upsampled to 512 × 768 pixels), and centered in a larger 1024 × 1024 pixel image where the remaining pixels were set to the mean luminance of each stimulus. In this ODOG implementation a 512 × 512 pixel image represents a square subtending 8° of visual angle, which matches the stimulus size in the perceptual experiments reported here.

Figure 9 shows the results, which are summarized in Table 1. ODOG makes qualitatively correct predictions for all individual stimuli in Figure 1, including those that show assimilation (Blakeslee & McCourt, 2004). However, as noted, comparisons with control conditions are also an important part of evaluating models, and here ODOG does less well. ODOG mistakenly predicts a stronger illusion in the argyle control condition than in the argyle illusion itself (as was shown by Kim et al., 2018). It also predicts a stronger illusion in the Koffka-connected control condition than in the Koffka-Adelson illusion. Furthermore, it predicts that the classic simultaneous contrast effect, which is relatively weak, is stronger than the argyle and snake illusions.

**Figure 10.**
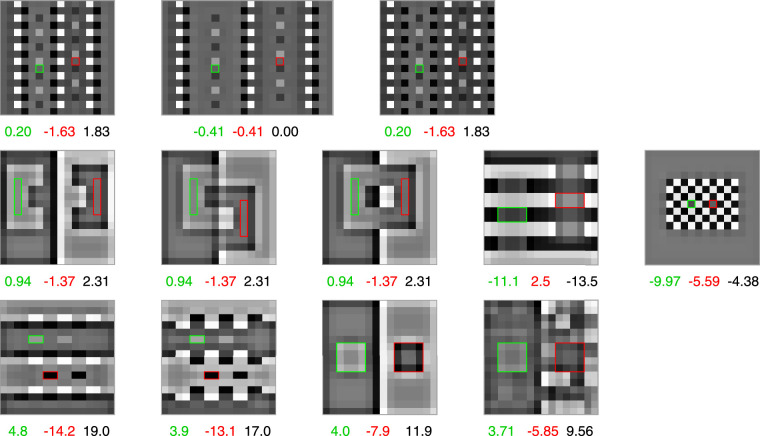
High-pass model results. See caption of Figure 9 for details.

Shapiro and Lu (2011) showed that several brightness illusions are predicted by a linear high-pass filter model. I tested this model on the same upsampled stimuli as ODOG. I tested a range of high-pass filter sizes (which is a parameter of Shapiro & Lu's model), and found that a filter that covered a 3 × 3 region in the 16 × 16 stimuli worked best. This corresponds to a 96 × 96 pixel filter in the upsampled stimuli.

Figure 10 and Table 1 show the results. The high-pass model makes qualitatively correct predictions for most of the individual stimuli in Figure 1, although it fails on stimuli that produce assimilation effects. The model also fails to handle control conditions correctly: In most cases, it predicts stronger illusions for control conditions than for the illusions themselves. It also mistakenly predicts that the classic simultaneous contrast effect is stronger than the argyle illusion.

**Figure 11.**
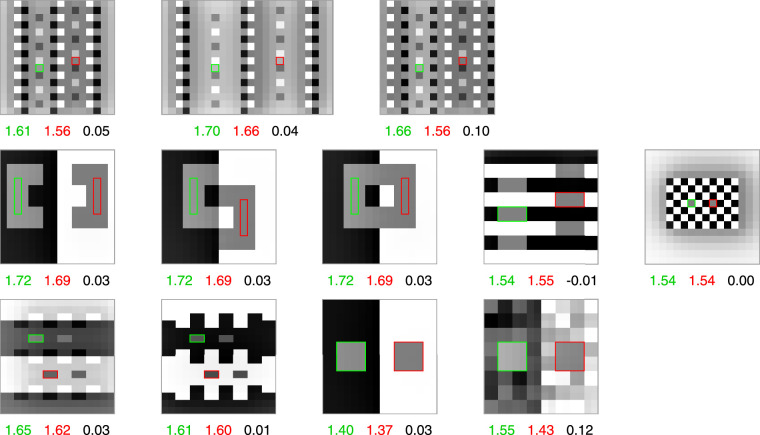
Retinex results. See caption of Figure 9 for details.

The high-pass model was originally designed to match its filter size to the target region in a stimulus (Shapiro & Lu, 2011), so I tested the high-pass model again with the filter size chosen separately for each stimulus. It was not always clear how to choose the filter size for the stimuli used here, so I simply chose plausible values. In these simulations, the high-pass model generally performed worse than reported in the previous paragraph. For the three argyle stimuli I used a 1 × 1 block (of the 16 × 16 stimuli) as the filter size, and the model predicted no illusion in any stimulus. For the three Koffka stimuli I used a 3 × 3 filter, and the model predicted the same illusion in the Koffka-connected control figure as in the Koffka-broken and Koffka-Adelson figures. For White's illusion I used a 2 × 2 filter, and for the checkerboard assimilation stimulus a 1 × 1 filter; in both cases the model predicted an illusion in the wrong direction. For the snake stimuli, I used a 1 × 1 filter, and the model predicted equally strong illusions in the snake and control conditions. For the simultaneous contrast stimuli I used a 4 × 4 filter; the model predicted the classic simultaneous contrast effect, but predicted a weaker illusion in the articulated figure. Thus, adapting the filter size to individual stimuli did not improve the model's performance.

McCann's (1999) retinex model is one of several revisions of Land and McCann's (1971) original model; see McCann and Rizzi (2012), chapter 12, for a review. McCann's model is a multiscale, coarse-to-fine algorithm that models visual appearance by propagating image luminance ratios over local regions. I tested Funt, Ciurea, and McCann's (2000) MATLAB implementation of the algorithm, with parameter nIteration set to 4. I upsampled the stimuli in the same manner as for ODOG and the high-pass model. Other retinex models operate differently from McCann's algorithm, so the results I report here test only this specific version of retinex.

Figure 11 and Table 1 show the results. The retinex model correctly predicts illusions in the individual stimuli in Figure 1, except those that show assimilation. It too fails to handle control conditions well, however, and predicts stronger illusions in the argyle and Koffka control stimuli than in the illusions themselves.

**Figure 12.**
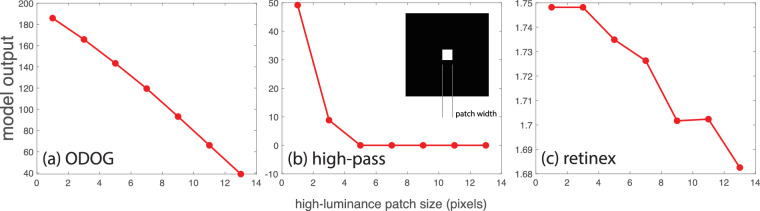
Model tests for glow perception. All three model outputs decrease as a function of test patch size, but none of the models has a criterion for what output range indicates reflective gray and what range indicates a light-emitting surface.

Overall, these results show that the individual illusions tested here are relatively easy to account for, except for assimilation effects, but that comparisons between illusions and their control conditions are more difficult. The argyle figure and its control condition are particularly difficult, possibly because the two target patches have identical immediate surrounds, and the illusion depends on the arrangement of more distant elements into X-junctions (Adelson, 1993). Thus, all three models tested correctly predict an illusion in the argyle figure but, unlike MIR, they predict at least as strong an illusion in the argyle control figure. Comparisons between the Koffka ring variants are also challenging, again possibly because of the role of X-junctions as evidence for lighting boundaries (Metelli, 1970; Beck et al., 1984).

I also tested whether these three models account for glow, codetermination, and articulation effects, using the same approach as for MIR (Figure 7). Tests for glow perception are shown in Figure 12, which plots model output as a function of the width of a central high-luminance patch. For all three models, the output for the central patch decreases as a function of patch width, as it does for MIR. However, these three models have no criteria for what output values correspond to reflective surfaces and what values indicate glow, so it is difficult to evaluate whether they predict perceived glow in such stimuli (cf. Economou et al., 2007). For the same reason, it is unclear how to test whether these three models account for the highest luminance rule (Figure 7a).

**Figure 13.**
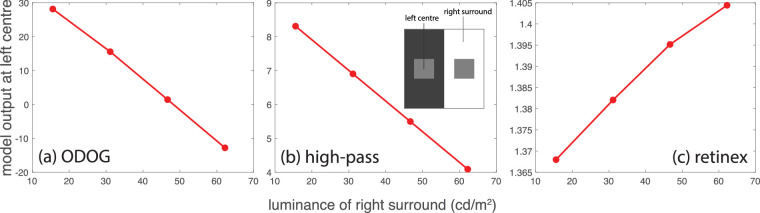
Model tests for codetermination. ODOG and the high-pass model predict qualitatively correct codetermination effects, but retinex does not.

Tests for codetermination are shown in Figure 13, which plots model output at the center left patch of a classic simultaneous contrast stimulus as a function of the luminance of the right-hand surround. ODOG and the high-pass model correctly predict codetermination effects, with left-center outputs that decrease as the right-surround luminance increases. Retinex shows a reverse codetermination effect, where the left-center output increases instead.

**Figure 14.**
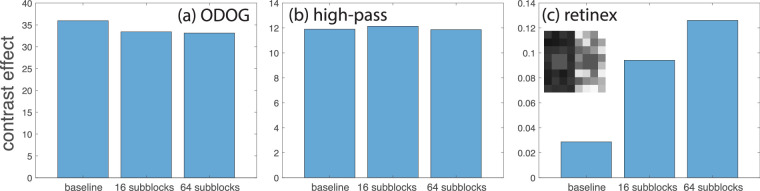
Model tests for articulation. Retinex predicts a qualitatively correct articulation effect, but ODOG and the high-pass model do not. The inset shows the 64 subblock stimulus.

Tests for articulation are shown in Figure 14, which plots the magnitude of a simultaneous contrast effect as the stimulus is divided into increasing numbers of subblocks. Retinex correctly predicts that the simultaneous contrast effect increases with greater articulation. The high-pass model predicts no effect, and ODOG predicts a small effect in the wrong direction.

## Discussion

Research on lightness perception has revealed several important principles regarding how we see achromatic images (e.g., the role of perceived lighting boundaries), but these findings have not usually been formulated as computational models. This often makes it difficult to know which principles are fundamental, and what the predictions are of several taken together. The present work takes a step toward addressing these problems. The modeling results reported here suggest, for example, that no special mechanisms are required to account for glow perception, and that the the straightness of lighting boundaries plays a central role in lightness perception despite the limited attention it has received in previous work (Logvinenko et al., 2005). Simple but precise models can make unexpected predictions (Geisler, 1984; Palmer et al., 2000), so there is much to be learned by formulating midlevel theories of lightness as computational models.

There is surprisingly little overlap between research on lightness and brightness, largely owing to differences in goals and methods. One difference is that current brightness models are almost always computational, whereas theories of lightness seldom are (although see the Introduction for some exceptions). This is a historical accident, however, and theories of lightness should aspire to be precise enough to make predictions for arbitrary images, just as brightness models do. Even low-level operations like convolution and contrast normalization may have promise for lightness models; ODOG accounts for an impressively wide range of stimuli with a relatively simple mechanism, and it seems entirely plausible that a model with similar elements could provide a physiologically motivated model of lightness that incorporates factors such as lighting boundaries (although see Betz et al., 2015). It may be that a Bayesian inference model like MIR describes lightness or brightness mechanisms at Marr's ‘computational’ level, whereas ODOG-like models describe them at the ‘implementation’ level (Marr, 1982).

Another difference is that brightness experiments usually show stimuli on computer monitors, whereas lightness experiments often use physical objects under real illumination. This difference may be more important than it seems. For example, classic experiments comparing lightness and brightness perception showed stimuli on computer monitors (Arend & Spehar, 1993; Blakeslee & McCourt, 2012). When judging lightness, observers were asked to report which stimulus regions appeared to be cut from the same piece of paper, but presumably no region of a light-emitting CRT in a dark room genuinely seemed to be cut from reflective paper. (The use of computer-generated stimuli may be one reason why some brightness researchers regard lightness judgments as partly “cognitive” rather than purely perceptual.) When judging brightness, observers were asked to report which stimulus regions had the same luminance, “disregarding, as much as possible, other areas of the display.” It is hard to say, however, how such instructions affect observers’ behavior or why brightness judgments should be more local than lightness judgments. One easy step toward a common theory of lightness and brightness would be to use stimuli where observers can make natural judgments of both properties; the most reliable way of doing this may be to use physical objects under realistic lighting, since lightness judgments, at least, can be very different with computer-generated and physical stimuli (Morgenstern, Geisler, & Murray, 2014; Patel, Munasinghe, & Murray, 2018; but see Radonjic et al., 2016).

The present work can be seen as a computational variant of anchoring theory (Gilchrist et al., 1999; Gilchrist, 2006). As with anchoring theory, a primary goal of MIR is to explain a wide range of qualitative lightness behaviors using a few general principles, rather than making a detailed model of a narrow range of tasks. MIR shows that several principles of anchoring theory – the highest luminance rule, codetermination, and so on – need not be posited as specific rules of lightness perception, because they emerge naturally from reasonable assumptions about scene statistics (Figure 7). Anchoring theory is sometimes presented as a theory of systematic lightness errors (Gilchrist et al., 1999), but if the present approach is correct then many of the behaviors it describes are actually consequences of the human visual system making rational use of statistical regularities in lighting and reflectance (Murray, 2013).

Another similarity to anchoring theory is that partial lighting boundaries play an important role in MIR. In anchoring theory, each local lighting framework has an associated weight that determines how strongly lightness estimates within the framework are affected by image luminances outside the framework; when a local framework is strongly segmented from the surrounding scene (e.g., by penumbra cues) the weight is low, and when it is weakly segmented the weight is high. Similarly, MIR interprets some luminance edges, such as those in the simultaneous contrast figure, as a combination of illuminance edges and reflectance edges (Figure 5i), and the CRF's potential functions place no additional cost on this interpretation. It is an interesting question how this behavior is related to the statistical properties of natural scenes, where such accidental alignments of lighting and reflectance edges are presumably rare. I leave this problem for future work, but it seems likely that, for some computational goals, hedging the interpretation as a combination of illuminance and reflectance edges will be an optimal solution, much as Bayesian cue combination models show that the optimal estimate of a scene property usually falls between the value indicated by a prior and the values indicated by cues (Maloney & Landy, 1989).

MIR is also consistent with psychophysical work showing that achromatic surfaces have at least two perceptual dimensions, namely, perceived reflectance and perceived illuminance (Logvinenko & Maloney, 2006; Logvinenko, 2015). One of the model's advantages is that it makes estimates of both these dimensions, which allows it to directly model the relationship between perceived lighting conditions and lightness estimates. Whether it can accommodate evidence against luminance-discounting models of lightness remains to be seen (Rutherford & Brainard, 2002).

As mentioned when describing the model, the statistical assumptions that guide MIR are tentative, and an important direction for improving the model will be to explore alternative assumptions. For example, does the model need an assumption that illuminance boundaries tend to be straight, given that a) the model puts a cost on illuminance edges and b) a straight line between two points is shorter than a curved line? The latter two properties would already seem to put a higher cost on curved illuminance boundaries. In fact, I have found that a variant of MIR without the straight illumination boundary assumption does account for effects such as the argyle and snake illusions (and their control conditions). However, it also creates artifacts where small reflectance regions are instead interpreted as lighting regions,^5^ and for this reason I have kept the straight illumination boundary assumption. To take another example, the assumption that low illuminances are more probable than high illuminances plays an important role in MIR, as it drives the model to assign reflectances that fill the range of 0.03 to 0.90 (MIR's version of the “highest luminance rule” in anchoring theory). It remains to be seen, however, whether this assumption correctly reflects natural scene statistics, or whether the human visual system's tendency to see the highest luminance in a scene as white has a different explanation (e.g., Murray, 2013). As these examples illustrate, there is a great deal of room for exploring the effects of alternative assumptions about lighting and reflectance. Another promising approach would be to learn potential functions from illuminance and reflectance patterns in natural scenes (or realistic renderings of natural scenes), instead of manually specifying a number of discrete assumptions (Freeman et al., 2000).

Finally, the CRF framework makes MIR highly extensible. Depth boundaries, for example, have a strong effect on lightness (Gilchrist, 1977), and if the model was provided with a depth map as an additional layer, it would be straightforward to reduce the cost of illuminance edges at depth discontinuities. Similarly, a model with three luminance, illuminance, and reflectance layers for three spectral bands, and with potential functions similar to those described here, would provide a starting point for a CRF model of color constancy (Land & McCann, 1971; Brainard & Wandell, 1986). It would also improve the model to incorporate factors such as grouping (Gilchrist, 2006) and haze (Adelson, 2000), and to allow naturalistic, high-resolution stimuli. Previous work on graphical models provides ample guidance and tools for such problems, and research on lightness perception, which often appeals to properties of lighting and reflectance in natural scenes, would benefit greatly from engaging with this literature.

## Figures and Tables

**Figure 15.**
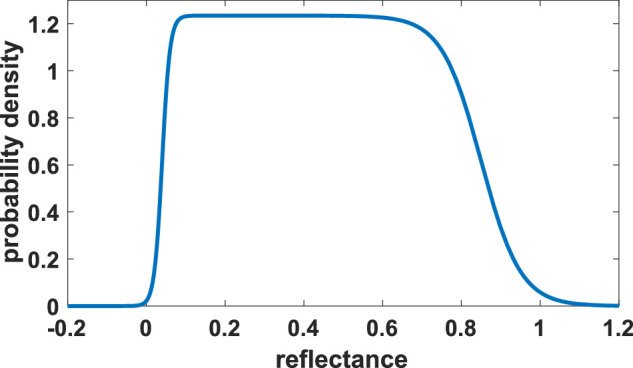
MIR's reflectance prior.

## Footnotes

1 Reflectance is the proportion of incident light reflected by a surface, e.g., black surfaces have a reflectance of around 3%. Lightness is *perceived reflectance*. Luminance is a measure of physical lighting magnitude in a viewed stimulus (units cd/m^2^). Brightness is *perceived luminance*. Illuminance is a measure of incident lighting magnitude on a surface (units lux).

2 An important detail is that this only holds if the MRF has *P*(*X*) > 0 for all *X*.

3 This is only a tendency, though, and exp (− ϵ_*i*_(*C*_*i*_)) is not proportional to the probability density of clique *C*_*i*_. Finding the marginal probability density of a clique from the Gibbs distribution of the ensemble (Equation 1) is not straightforward.

4 This aspect of the cost functions is explained in detail in the Appendix. The cost functions ϵ_*i*_ are defined on 2 × 2 cliques, so a more complete statement of this property is that cost functions are evaluated on uniform image regions, up to a maximum 2 × 2 pixel region.

5 In the MATLAB implementation of MIR (doi:10.17605/OSF.IO/4FWJV), the reader can set the parameter model.wmtwist (which controls the cost of curved illuminance boundaries) to zero to see these effects.

## Appendix

### The Markov illuminance and reflectance model

#### Model structure

The Markov illuminance and reflectance (MIR) model is a CRF with two 16 × 16 layers. Layer 1 represents the observed luminance image *L* = (*l*_*ij*_), and layer 2 represents the illuminance assignment *M* = (*m*_*ij*_). For a Lambertian, frontoparallel surface, these two layers imply a reflectance assignment $R=\left(r_{ij}\right)=\left(\pi \;l_{ij}/m_{ij}\right)$. (The factor of π arises when we use units of cd/m^2^ for luminance and lux for illuminance.) Each node in layer 2 (except edge nodes) has an undirected connection to the nearest nine neighbors in layer 1 and the eight nearest neighbors in layer 2. Given an observed luminance image *L*, the model represents the posterior probability density of illuminance *M* and implied reflectance *R* as a Gibbs distribution, with total energy given by a sum of local potentials over 4-cliques (i.e., 2 × 2 squares) in layer 2:

(2) $$P\left(M,R|L\right)=\frac{1}{Z}\;\text{exp}\left(-\sum_{ij}\varepsilon_{ij}\left(M_{ij};L\right)\right)$$

Here, *ij* indexes the 4-clique with its upper left corner in row *i* and column *j*. ϵ_*ij*_ is the potential function that returns the energy of the 4-clique *ij* (see Section 2 for details). *M*_*ij*_ is the 2 × 2 submatrix of *M* on the 4-clique *ij*. *Z* is the partition function, a constant that normalizes the probability density to unit volume.

Layer 2 explicitly represents illuminance assignments *m*_*ij*_, but some of its potential functions (see below) also include costs that depend on the implied reflectance $r_{ij}=\pi \;l_{ij}/m_{ij}$. Thus, a more complete way of describing layer 2 is to say that it represents illuminance-reflectance assignments (*m*_*ij*_, *r*_*ij*_), parameterized by the illuminance *m*_*ij*_. In the exposition in the main text (e.g., Figure 2), I gave the two layers equal status for simplicity, but in fact the illuminance layer *M* is represented explicitly, and the reflectance layer *R* is implied.

#### Potential functions

The model uses the following soft constraints, implemented via the potential functions ϵ_*ij*_ in Equation 2. These are the seven assumptions described in the main text.

(A1) *Illuminance spans a wide range, and lower illuminances are more likely.* Each node in layer 2 has a potential $\varepsilon_{ij}^{1m}=\log_{10}m_{ij}$ that assigns a logarithmic cost to the illuminance *m*_*ij*_ at that node. This allows illuminance to take any positive value, but assigns a lower cost, and hence a higher probability, to lower values.

I label potential functions ϵ with superscripts. In $\varepsilon_{ij}^{1m}$, the 1 superscript indicates that this is a potential on a 1-clique (a single node), and the *m* indicates that it places a cost on illuminance. In the accompanying MATLAB implementation, $\varepsilon_{ij}^{1m}$ (and all other potential functions) are represented as a table, and *m*_*ij*_ takes 20 logarithmically spaced values between 30 lux and 350 lux.

(A2) *Reflectance mostly spans the range of 3% to 90%.* Reflectance is modeled using the following probability density function, which is shown in Figure 15:

(3) $$\begin{array}{ccc} f_{r}\left(r\right) & = & \frac{1}{0.85-0.04}\min(\sigma \left(r;0.04,0.01\right),\\ & & \;\;\;\;\;\sigma (-r;-0.85,0.05))\; \end{array}$$

Here, $\sigma \left(x;a,b\right)={\left(1+e^{\frac{-(x-a)}{b}}\right)}^{-1}$ is the logistic function. *f*_*r*_ is the minimum of an increasing and a decreasing logistic function, with parameters *a* and *b* chosen so that most of the area under the resulting function lies between *r* = 0.03 and *r* = 0.90. Each node in layer 2 has a potential $\varepsilon_{ij}^{1r}=-\log_{10}f_{r}\left(\pi \;l_{ij}/m_{ij}\right)$, the negative log likelihood of the implied reflectance $r_{ij}=\pi \;l_{ij}/m_{ij}$.

MIR is robust with respect to changes in the reflectance density function in Equation 3. For example, the model gives qualitatively similar results with a density function that takes a constant value between 0.03 and 0.90, and a value of 10^−5^ elsewhere. The key properties of the density function are that it mostly limits reflectance to a physically realistic range, and allows values outside this range with some small probability.

For later convenience (see (A6)), I group the 1-clique potentials $\varepsilon_{ij}^{1m}$ and $\varepsilon_{ij}^{1r}$ into 2-clique potentials. The horizontal 2-clique indexed by *ij* is the 1 × 2 block of layer 2 nodes with its left node at position *ij*. The vertical 2-clique indexed by *ij* is the 2 × 1 block with its upper node at position *ij*. I define horizontal and vertical 2-potentials (with superscript labels *h* and *v*) as follows.

(4) $$\varepsilon_{ij}^{2hmr}=\frac{\varepsilon_{ij}^{1m}+\varepsilon_{ij}^{1r}}{k_{ij}}+\frac{\varepsilon_{i(j+1)}^{1m}+\varepsilon_{i(j+1)}^{1r}}{k_{i(j+1)}}$$

(5) $$\varepsilon_{ij}^{2vmr}=\frac{\varepsilon_{ij}^{1m}+\varepsilon_{ij}^{1r}}{k_{ij}}+\frac{\varepsilon_{(i+1)j}^{1m}+\varepsilon_{(i+1)j}^{1r}}{k_{(i+1)j}}$$

Here, *k*_*ij*_ is the number of horizontal or vertical 2-cliques that 1-clique *ij* belongs to, which is four except at edge nodes. Dividing by *k*_*ij*_ distributes the 1-clique potentials evenly, in such a way that summing all 2-clique potentials $\varepsilon_{ij}^{2hmr}$ and $\varepsilon_{ij}^{2hmr}$ gives the same result as summing all 1-clique potentials $\varepsilon_{ij}^{1m}$ and $\varepsilon_{ij}^{1r}$.

In Equations 4 and 5, and elsewhere, I leave implicit the fact that potentials for different clique sizes have different index ranges *ij*. For 1-clique potentials $\varepsilon_{ij}^{1*},$ the range is *i* = 1: 16, *j* = 1: 16. For horizontal 2-clique potentials $\varepsilon_{ij}^{2h*},$ the range is *i* = 1: 16, *j* = 1: 15, because $\varepsilon_{i,16}^{2h*}$ would extend beyond the right side of the CRF. Similarly, for vertical 2-clique potentials $\varepsilon_{ij}^{2v*},$ the range is *i* = 1: 15, *j* = 1: 16, and for 4-clique potentials $\varepsilon_{ij}^{4*},$ the range is *i* = 1: 15, *j* = 1: 15.

(A3) *Illuminance edges are less common than reflectance edges.* Each horizontal and vertical 2-clique has a potential that assigns a sum-of-squares cost to log-illuminance edges:

(6) $$\varepsilon_{ij}^{2hm}=w\;{\left(\log m_{ij}-\log m_{i(j+1)}\right)}^{2}$$

(7) $$\varepsilon_{ij}^{2vm}=w\;{\left(\log m_{ij}-\log m_{(i+1)j}\right)}^{2}$$

The weight *w* depends on the local image luminance pattern as described in (A4) and (A5). The weights were arrived at by manual adjustment, and the model is fairly flexible with regard to weight assignments. The model assigns no cost to reflectance edges.

(A4) *X-junctions are evidence for illuminance edges.* For a two-clique at an image luminance edge that is part of an X-junction, the potentials in Equations 6 and 7 have zero weight, *w* = *w*_*x*_ = 0. I liberally define an X-junction as a 2 × 2 square where there are luminance edges between all four pairs of adjacent nodes; no special relationship between the four luminances is required. In future revisions of the model, it will be worth exploring whether requiring physically realistic luminance relationships in X-junction cues to lighting boundaries improves performance (Metelli, 1970).

(A5) *Illuminance edges tend to occur at image luminance edges.* For a 2-clique at an image luminance edge that is not part of an X-junction, the potentials in Equations 6 and 7 have a moderate weight, *w* = *w*_1_ = 20. For a 2-clique with no luminance edge, the potentials have a large weight, *w* = *w*_0_ = 600. These and the other weights *w*_*_ in the model were arrived at by manual experimentation.

(A6) *Potentials are evaluated on uniform image regions, not pixelwise.* Articulation effects show that lightness constancy tends to be better in images that consist of many distinct luminance regions (Katz, 1935). One possible explanation for these effects is that the visual system partitions an image into uniform luminance regions, and considers each region to provide a sample of information about the lighting conditions in the scene: more luminance regions provide more information, and as a result lightness percepts tend to be more accurate. I cannot fully implement this hypothesis in the present model, as potentials are evaluated over regions no larger than 4-cliques. I take a step in this direction, though, by having 2-clique potentials that span a luminance edge provide twice the potential of 2-clique potentials that fall within uniform luminance regions. I could do the same with 4-clique potentials, but I find that modifying 2-clique potentials this way is sufficient to create articulation effects.

I define the following horizontal and vertical 2-clique potentials that combine the 2-clique potentials defined above, and divide them by two if they fall within a uniform-luminance region.

(8) $$\begin{array}{ccc} \varepsilon_{ij}^{2h} & = & \frac{\varepsilon_{ij}^{2hmr}+\varepsilon_{ij}^{2hm}}{{a\;}_{ij}^{h}}\;\text{where}\;\\ {a\;}_{ij}^{h} & = & \left\{\begin{array}{cc} 2 & \text{if}\;\;l_{ij}=l_{i(j+1)}\\ 1 & \text{otherwise} \end{array}\right. \end{array}$$

(9) $$\begin{array}{ccc} \varepsilon_{ij}^{2v} & = & \frac{\varepsilon_{ij}^{2vmr}+\varepsilon_{ij}^{2vm}}{a_{ij}^{v}}\;\text{where}\;\\ a_{ij}^{v} & = & \left\{\begin{array}{cc} 2 & \text{if}\;\;l_{ij}=l_{(i+1)j}\\ 1 & \text{otherwise} \end{array}\right. \end{array}$$

(A7) *Illuminance edges tend to be straighter than reflectance edges.* Each 4-clique has a potential $\varepsilon_{ij}^{4m}$ that assigns a cost to any 2 × 2 illuminance pattern that is not a multiplicative combination of vertical and horizontal edges. Consider a local illuminance pattern:

(10) $$\left[\begin{array}{cc} m_{11} & m_{12}\\ m_{21} & m_{22} \end{array}\right]$$

If the pattern is a horizontal edge, for example,

(11) $$\left[\begin{array}{cc} 1 & 1\\ 2 & 2 \end{array}\right]$$

then *m*_11_/*m*_21_ = *m*_12_/*m*_22_, or alternatively τ ≡ (log_10_*m*_11_ − log_10_*m*_21_) − (log_10_*m*_12_ − log_10_*m*_22_) = 0. If the pattern is a vertical edge, for example,

(12) $$\left[\begin{array}{cc} 1 & 2\\ 1 & 2 \end{array}\right]$$

then *m*_11_/*m*_12_ = *m*_21_/*m*_22_, and again τ = 0. Any pointwise product of horizontal and vertical edges also has τ = 0. Thus, a 2 × 2 illuminance pattern produced by a vertical or horizontal shadow, or by a shadow meeting a transmissive filter at right angles (Metelli, 1970), or by two transmissive filters meeting at right angles under uniform illumination, all have τ = 0.

I call τ the *twist* of the log illuminance, as it is a discrete analog to $\frac{\partial^{2}}{\partial x\partial y}\log_{10}m\left(x,y\right)$ and measures the local rate of slope change in log illuminance. There are many ways of quantifying the straightness of local illuminance edges, but I have found τ to be an easy-to-compute and effective measure. The potential $\varepsilon_{ij}^{4m}$ puts a cost on τ:

(13) $$\begin{array}{ccc} \varepsilon_{ij}^{4m} & = & 200\;\tau_{ij}^{2}=200[\log_{10}m_{ij}+\log_{10}m_{\left(i+1)(j+1\right)}\\ & & -\log_{10}m_{(i+1)j}-\log_{10}m_{i(j+1)}{]}^{2} \end{array}$$

The scale factor 200 is a manually chosen value that makes the model produce human-like interpretations of lightness illusions. The model puts no cost on the twist of reflectance.

The full potential ϵ_*ij*_ for each 4-clique (see Equation 2) is the sum of the twist potential and the 2-potentials contained within the 4-clique:

(14) $$\varepsilon_{ij}=\varepsilon_{ij}^{4m}+\frac{\varepsilon_{ij}^{2h}}{k_{ij}^{2h}}+\frac{\varepsilon_{(i+1)j}^{2h}}{k_{(i+1)j}^{2h}}+\frac{\varepsilon_{ij}^{2v}}{k_{ij}^{2v}}+\frac{\varepsilon_{i(j+1)}^{2v}}{k_{i(j+1)}^{2v}}$$

Here $k_{ij}^{2h}$ is the number of 4-cliques that horizontal 2-clique *ij* belongs to, which is two except in the top and bottom rows of the CRF. $k_{ij}^{2v}$ is the corresponding number for vertical 2-cliques. Dividing by $k_{ij}^{2h}$ and $k_{ij}^{2v}$ distributes the 2-clique potentials evenly, in such a way that summing all 4-clique potentials ϵ_*ij*_ gives the same result as summing all $\varepsilon_{ij}^{4m}$, $\varepsilon_{ij}^{2h}$, and $\varepsilon_{ij}^{2v}$.

#### Inference

The model uses max-sum belief propagation to estimate the maximum a posteriori (MAP) assignment of illuminance and reflectance to a luminance image. The strictly correct form of the max-sum algorithm is impractical with densely connected graphical models such as the one used here, but with some heuristics it can be made to work well. Here I implement belief propagation using a Bethe cluster graph (e.g., [p. 405] Koller & Friedman, 2009) and tree reparameterization (e.g., [p. 408] Koller & Friedman, 2009).

The Bethe cluster graph for this model has two kinds of nodes: 1-nodes that represent single CRF nodes in layer 2, and 4-nodes that represent the potential function ϵ_*ij*_ for each 4-clique in layer 2. There is an undirected connection between each 1-node and the 4-nodes that represent the potential functions that the 1-node contributes to. In each message passing operation, a node in the cluster graph sends a function δ(*m*_*ij*_) to another node that it is connected to. The max-sum message δ(*m*_*ij*_) reports the lowest energy of any illuminance-reflectance assignment to the sending node's clique, that assigns illuminance *m*_*ij*_ (and so reflectance $r_{ij}=\pi \;l_{ij}/m_{ij}$) to the one CRF node *ij* that the sender and receiver have in common. The message takes into account incoming messages from the sending node's other neighbors, and if the sending node is a 4-node then it also takes into account the node's potential ϵ_*ij*_.

I use tree reparameterization to choose the message passing schedule. The Bethe cluster graph has 60 horizontal and vertical subtrees. To make one complete message passing iteration, I randomly permute the order of these subtrees, and do an upward-downward message passing sweep on each subtree. I find this method to be much more effective than choosing individual nodes in random order.

Max-sum belief propagation is guaranteed to converge to a global energy minimum when the cluster graph it operates on has no loops. The Bethe cluster graph used here has many loops, so the algorithm is not guaranteed to converge, and when it does converge, it may not converge to a global minimum. Nevertheless, max-sum belief propagation is often found to work well in practice on cluster graphs with loops, and that is what I find here, particularly in conjunction with tree reparameterization. The algorithm practically always converges to a reasonable illuminance-reflectance assignment, though usually not to the global optimum, and sometimes with artifacts such as illuminance edges at unexpected locations. I improve convergence by taking the best (lowest-energy) result of ten independent runs of belief propagation, each consisting of five complete iterations as defined above in the paragraph on tree reparameterization. After 10 runs, further runs do not usually find substantially better solutions.
